# First-year mortality in incident dialysis patients: results of the Peridialysis study

**DOI:** 10.1186/s12882-022-02852-1

**Published:** 2022-06-27

**Authors:** James Heaf, Maija Heiro, Aivars Petersons, Baiba Vernere, Johan V. Povlsen, Anette Bagger Sørensen, Naomi Clyne, Inga Bumblyte, Alanta Zilinskiene, Else Randers, Niels Løkkegaard, Mai Rosenberg, Stig Kjellevold, Jan Dominik Kampmann, Björn Rogland, Inger Lagreid, Olof Heimburger, Abdul Rashid Qureshi, Bengt Lindholm

**Affiliations:** 1 Zealand University Hospital, Roskilde, Denmark; 2 Turku University Hospital, Turku, Finland; 3 P. Stradins University Hospital, Rīga, Latvia; 4 Aarhus University Hospital, Aarhus, Denmark; 5 Lund University and Skåne University Hospital, Lund, Sweden; 6 Lithuanian University of Health Sciences, Kaunas, Lithuania; 7 Viborg Regional Hospital, Viborg, Denmark; 8 Holbaek Hospital, Holbæk, Denmark; 9 Tartu University and University Hospital of Tartu, Tartu, Estonia; 10 Vestfold Hospital, Tønsberg, Norway; 11 Hospital of Southern Jutland, Soenderborg, Denmark; 12 Kristianstad Hospital, Kristianstad, Sweden; 13 St, Olav University Hospital, Trondheim, Norway; 14 Karolinska Institutet, Stockholm, Sweden

**Keywords:** Hemodialysis, Peritoneal dialysis, Survival analysis, Mortality

## Abstract

**Background:**

Controversy surrounds which factors are important for predicting early mortality after dialysis initiation (DI). We investigated associations of predialysis course and circumstances affecting planning and execution of DI with mortality following DI.

**Methods:**

Among 1580 patients participating in the *Peridialysis* study, a study of causes and timing of DI, we registered features of predialysis course, clinical and biochemical data at DI, incidence of unplanned suboptimal DI, contraindications to peritoneal dialysis (PD) or hemodialysis (HD), and modality preference, actual choice, and cause of modality choice. Patients were followed for 12 months or until transplantation. A flexible parametric model was used to identify independent factors associated with all-cause mortality.

**Results:**

First-year mortality was 19.33%. Independent factors predicting death were high age, comorbidity, clinical contraindications to PD or HD, suboptimal DI, high eGFR, low serum albumin, hyperphosphatemia, high C-reactive protein, signs of overhydration and cerebral symptoms at DI. Among 1061 (67.2%) patients who could select dialysis modality based on personal choice, 654 (61.6%) chose PD, 368 (34.7%) center HD and 39 (3.7%) home HD. The 12-months survival did not differ significantly between patients receiving PD and in-center HD.

**Conclusions:**

First-year mortality in incident dialysis patients was in addition to high age and comorbidity, associated with clinical contraindications to PD or HD, clinical symptoms, hyperphosphatemia, inflammation, and suboptimal DI. In patients with a “free” choice of dialysis modality based on their personal preferences, PD and in-center HD led to broadly similar short-term outcomes.

## Introduction

The survival in patients with end stage kidney disease (ESKD) starting on dialysis therapy has improved during recent years but the mortality remains high, especially during the initial months on dialysis therapy [1–4]. There are many potential biological factors contributing to the poor initial outcomes [5, 6]. In incident hemodialysis (HD) patients, use of central venous catheter and hypoalbuminemia are associated with the highest early mortality risk [7]. In addition to biological factors, late versus early nephrologist referral, predialysis care, and circumstances of dialysis initiation (DI), especially suboptimal DI, may play a role.

*Early referral* to specialist nephrological care is associated with reduced mortality after DI [8–13]. It is possible that this permits early dialysis planning with identification of patients who wish to have peritoneal dialysis, timely AVF placement for those who wish to have hemodialysis, and early identification of patients with rapid uraemia progression. Rapid loss of renal function prior to DI, which is more common in HD than in PD patients [14], is related to increased mortality [15].

*Predialysis care* at a multidisciplinary predialysis clinic is associated with reduced mortality [16–19]. Possible causality is speculative, putative factors being better patient attention to symptoms, improved dietary and therapeutic treatment [19] and reduced frequency of suboptimal DI.

*Suboptimal DI* is associated with increased mortality [7, 20–24]. This may be causal, e.g., due to increased incidence of bacteraemia compared to use of arteriovenous fistula (AVF) [25], but may just be a marker of the acute morbidity that often requires suboptimal DI.

*The Peridialysis study* is a multi-center international observational study allowing retrospective analyses of prospectively collected data of the relevance of pre-dialysis renal care for causes of DI, timing of DI, modality choice, and clinical outcomes [26–28]. Among major advantages that distinguishes the Peridialysis study in comparison to previous studies it should be noted that data was registered prospectively in patients who were assessed before or at DI for suitability for HD and PD, and that the reason for choice of modality after assessment of suitability was registered. This has not previously been studied as a confounding factor.

The present study focuses on the relationships of the predialysis course, the clinical and biochemical circumstances at DI, and choice of modality, on short-term (1-year) mortality after DI. Three different models were studied, one concerning the predialysis course, one the biochemical variables at first dialysis, and one the clinical situation at dialysis. Finally, a combined model of all factors was studied.

## Materials and methods

This observational multinational multi-centre study comprised 1619 ESKD patients who started dialysis over a 3-year period at 15 nephrology departments from seven Nordic and Baltic countries. The methodology of the Peridialysis project has been previously described [26–28]. All centers delivered both PD and in-centre HD; some also home-HD; all had a developed and working multidisciplinary pre-dialysis care team structure with nephrologists and experienced nurses; 13/15 centers also had access to a dietician, and 5/15 had access to social worker.

The commonest method of assessing residual renal function and guiding clinical treatment was estimated glomerular filtration rate (eGFR) as measured by the Chronic Kidney Disease Epidemiology Collaboration (CKD-EPI) formula [29].

### Patients

Patients included in this study were consecutive patients starting chronic dialysis therapy for ESKD at the participating centers between January 1, 2015 and December 31, 2017. Five centers had a shorter recruiting period.

A patient was considered as having ESKD at first dialysis if:

1. The patient was diagnosed as having ESKD (thus excluding patients with acute kidney failure) according to the treating physician; this was the most used definition of ESKD.
2. The patient received dialysis treatment for > 90 days.
3. If the doctor was in doubt whether the patient had acute or chronic renal failure, the patient was included retrospectively as soon as there was no doubt that the patient had chronic renal failure and ESKD.

All methods were carried out in accordance with relevant guidelines and regulations. The study protocol was approved by the ethical review boards in centers located in countries where according to the country´s regulations this was required. The study was approved by the Swedish Ethical Review Authority (Ref 2017/7), while in Denmark, due to the observational non-interventional design of the study using anonymized patient data, the study protocol was not considered to be eligible for ethical review. Informed consent—either written or verbal depending on the regulations in the different countries—was obtained from participants in all centers including those in Denmark, with the exception of Lithuania, where patient permission was waived by the ethics board (P2-BE-2–9/2014). The study is registered with Clinical Trials.gov, identifier NCT02488200. The Swedish approval was valid for all EU countries.

## Methods

### Patient clinical data

The following data were registered at DI: patient characteristics (age, sex, height, body weight, body mass index (BMI) and underlying renal diagnosis), selected comorbidities (previous myocardial infarction, heart failure, cardiac atherosclerosis, cerebrovascular disease, diabetes, peripheral atherosclerosis, previous cancer (except basocellular), chronic pulmonary disease, chronic liver disease, psychiatric disease, and “other chronic conditions” and previous renal transplantation. Data on initial dialysis access was used to classify if the start of dialysis as optimal DI or suboptimal DI.

DI was classified as optimal if:

1. The access was an AVF or graft (AVG);
2. The access was a tunnelled vascular catheter to be used as the patient’s permanent access due to a medical decision;
3. The access was a PD catheter, and PD was started > 6 days after placement.

DI was classified as suboptimal if:

1. The access was a temporary vascular catheter;
2. The access was a tunnelled catheter, but a later AVF/AVG was planned;
3. The access was a PD catheter, and PD was started < 6 days after placement.

Late referral was defined as referral to the specialist clinic < 3 months before DI.

As pre-emptive transplants were often assessed and treated at other departments, patients receiving pre-emptive transplants were excluded from the study.

Multidisciplinary predialysis care and use of shared decision making with involvement of patients and their family and the renal team was routine in the centres involved in this study [26–28].

Modality choice was planned by shared decision making before DI or shortly thereafter. If either HD or PD were a priori contraindicated, the cause of modality choice was categorized considering the presence of the specific reasons for choosing one or the other of the two modalities: “PD (HD contraindicated or not possible)” or “HD (physical contraindication to PD)”, “HD (mental contraindication to PD)” or “HD (abdominal contraindication to PD)”. Patients with whom the possibility of home dialysis modalities was not assessed or discussed were registered as “HD (home dialysis modality not discussed)”. The remaining patients, i.e., patients with a “free” choice of dialysis modality based on their personal preferences, could choose between PD, in-centre HD and home HD after receiving information about the modalities as described previously [26–28]. Changes in modality during the first year after DI were registered.

### Biochemical data

The following biochemical data prior to or in conjunction with first dialysis were registered: blood hemoglobin, plasma concentrations of urea, creatinine, potassium, hydrogen carbonate (bicarbonate), albumin, C-reactive protein (CRP), total or ionized calcium, and phosphate. Most centers measured ionized calcium; for other centers, ionized calcium was assumed to be 50% of total calcium. Whenever available, plasma creatinine concentration and date of measurement were registered about three and six months before DI.

For patients whose eGFR had been determined approximately three months before DI, rate of change of eGFR during the three months before DI was calculated. Rapid rate of loss of eGFR was defined as a fall of eGFR > 1 ml/min/1.73m^2^/month.

### Reasons for dialysis initiation stated in questionnaire to physicians

Physicians gave details in an English language questionnaire of their reasons for prescribing chronic dialysis at DI. They could choose between several pre-stated clinical and/or biochemical reasons. Details of these have already been published [26–28]. For the purposes of the present study, clinical symptoms were registered if they were the primary cause of DI. Life-threatening conditions were defined as presence of pulmonary stasis, dyspnoea, cardiac symptoms, pericarditis, acidosis or hyperkalemia. Clinical reasons (rather than biochemical) were stated to be the primary cause of DI in 63% % of patients [26].

### Statistics

Data are expressed as median (IQR, interquartile range) or percentage. Statistical significance was set at the level of *p* < 0.05. *P* values were not adjusted for multiple testing [30]. Comparisons between two groups were assessed with the non-parametric Wilcoxon test for continuous variables and Chi-square test for nominal variables. Kaplan–Meier survival curves were used for univariate analyses of overall survival.

A flexible parametric survival model with stpm2 command [31] was used to identify independent factors associated with mortality risk, expressed as hazard ratios, during the first year of dialysis. The flexible parametric model was used instead of the Cox proportional hazards model because the assumptions of the latter that hazards were proportional and constant over time were not fulfilled. Patients were censored for lost-to-follow-up or renal transplantation. In all analyses, adjustments were made for age, sex, comorbidity, and renal diagnoses, and in addition for selected variables as follows: the “Predialysis” model included predialysis variables: rate of eGFR change per month prior to DI, suboptimal DI, and modality choice. The “Biochemical” model included selected biochemical variables at DI: hemoglobin, plasma concentrations of urea, creatinine, potassium, hydrogen carbonate (bicarbonate), albumin, CRP and total or ionized calcium, and phosphate. The “Clinical” model included clinical problems that were stated as the primary cause of DI: pulmonary stasis, dyspnea, cerebral symptoms, edema, cardiac symptoms, fatigue, and anorexia. Finally, a “Combined model” included all factors that were statistically significant in the previous three models.

Statistical analyses were performed using statistical software SAS version 9.4 (SAS Campus Drive, Cary, NC, USA) and Stata 17.0 (Stata Corporation, College Station, TX, USA).

## Results

Altogether 1619 patients were recruited to the study; 39 patients were excluded due to insufficient basic data (*n* = 10), lack of follow-up (*n* = 12) or pre-emptive transplantation (*n* = 17). The remaining 1580 patients (age 63.7 ± 15.4 years; women 35.9%; eGFR at DI on average 7.3 ± 3.6, median 6.7 (IQR 5.0–8.5) ml/min/1.73m^2^) were included in the present study. Clinical details at DI were available for 1544 (97.7%) patients and biochemical data in 1533 (97.0%) patients. The patient details of all patients and when divided according to their initial dialysis treatment, PD and HD, are shown in Table 1. Patients starting on PD had higher eGFR, less comorbidity, less abnormal biochemical status, and clinical symptoms representing primary cause of DI were in general less severe.

**Table 1:** Clinical and laboratory characteristics in all 1580 patients initiating dialysis and when divided based upon initial therapy of PD or HD

	**All**	**PD**	**HD**	***p*-value**
	***N* = 1,580**	***N* = 561**	***N* = 1,019**	
Age, years	67.0 (54.4–74.7)	66.3 (53.5–74.4)	67.3 (55.2–75.0)	0.20
Males	1,012 (64.1%)	363 (64.7%)	649 (63.7%)	0.69
BMI, kg/m^2^ (*n* = 1369)	25.8 (22.9–29.4)	25.8 (23.0–28.7)	25.8 (22.8–30.0)	0.26
Albumin, g/L (*n* = 1412)	33.0 (28.0–37.9)	34.9 (30.0–38.0)	32.0 (27.0–37.0)	< 0.001
eGFR epi ml/min1.73 m^2^	6.7 (5.0–8.5)	7.2 (5.7–9.3)	6.4 (4.7–8.2)	< 0.001
High eGFR loss rate^a^ n(%)	527 (43.4%)	134 (28.3%)	393 (53.1%)	< 0.001
**Renal diagnosis**				
Diabetic nephropathy	386 (24.4%)	145 (25.8%)	241 (23.7%)	0.33
Polycystic kidney disease	105 ( 6.6%)	45 ( 8.0%)	60 ( 5.9%)	0.10
Glomerulonephritis	283 (17.9%)	116 (20.7%)	167 (16.4%)	0.033
Chronic interstitial nephritis	186 (11.8%)	51 ( 9.1%)	135 (13.2%)	0.014
Hypertensive nephropathy	301 (19.1%)	117 (20.9%)	184 (18.1%)	0.18
**Comorbidity**				
Previous myocardial infarct	170 (10.8%)	57 (10.2%)	113 (11.1%)	0.57
Heart failure	262 (16.6%)	89 (15.9%)	173 (17.0%)	0.57
Other heart disease	196 (12.4%)	59 (10.5%)	137 (13.4%)	0.091
Cerebrovascular	188 (11.9%)	60 (10.7%)	128 (12.6%)	0.27
Clinical diabetes mellitus	548 (34.7%)	196 (34.9%)	352 (34.5%)	0.87
Peripheral atherosclerosis	193 (12.2%)	57 (10.2%)	136 (13.3%)	0.064
Cancer	261 (16.5%)	73 (13.0%)	188 (18.4%)	0.005
Pulmonary	150 ( 9.5%)	44 ( 7.8%)	106 (10.4%)	0.097
Hepatic	60 ( 3.8%)	19 ( 3.4%)	41 ( 4.0%)	0.53
Previous transplant	81 ( 5.1%)	22 ( 3.9%)	59 ( 5.8%)	0.11
Psychiatric	67 ( 4.2%)	14 ( 2.5%)	53 ( 5.2%)	0.011
**Biochemical**				
Hemoglobin, ref ≥ 7 mmol/L	1,142 (73.1%)	336 (61.0%)	806 (79.7%)	< 0.001
Urea, ref = < 30 mmol/L	876 (57.1%)	230 (42.7%)	646 (65.0%)	< 0.001
Potassium < 5 ref	414 (26.8%)	93 (17.0%)	321 (32.2%)	< 0.001
Bicarbonate ref = < 15 mmol/L	1,048 (89.3%)	393 (97.5%)	655 (85.1%)	< 0.001
Ionized calcium ≥ 1.15 mmol/L	769 (51.2%)	331 (62.0%)	438 (45.3%)	< 0.001
Phosphate ≥ 2.0 mmol/L	654 (44.2%)	171 (32.0%)	483 (51.2%)	< 0.001
**Clinical complications at DI**				
Pulmonary stasis	126 ( 8.2%)	22 ( 4.0%)	104 (10.5%)	< 0.001
Dyspnea	70 ( 4.5%)	18 ( 3.3%)	52 ( 5.2%)	0.075
Cerebral symptoms	16 ( 1.0%)	1 ( 0.2%)	15 ( 1.5%)	0.014
Edema	117 ( 7.6%)	39 ( 7.1%)	78 ( 7.9%)	0.58
Cardiac symptoms	36 ( 2.3%)	14 ( 2.5%)	22 ( 2.2%)	0.68
Fatigue	293 (19.0%)	128 (23.2%)	165 (16.6%)	0.001
Anorexia	227 (14.7%)	104 (18.9%)	123 (12.4%)	< 0.001
**Mortality rate**^**b**^				
0 – 3 months, n(%)	108(23.90)	23(14.11)	85(29.42)	0.001
3 – 6 months, n(%)	78(18.86)	22(14.15)	56(21.37)	0.12
6 – 9 months, n(%)	68(17.96)	27(19.59)	41(17.96)	0.56
9 – 12 months, n(%)	53(15.43)	20(16.10)	33(15.02)	0.80
0 – 12 months, n(%)	307(19.33)	92 (15.95)	215(21.26)	0.02

Data are presented as n (%) for categorial measures and median (IQR, interquartile range) for continuous variables

^a^eGFR loss > 1 ml/min1.73m^2^/month during 3 to 0 months prior to DI /month

^b^n (%), Number of deaths (deaths per 100 patient-years for each interval)

**Fig. 1:**
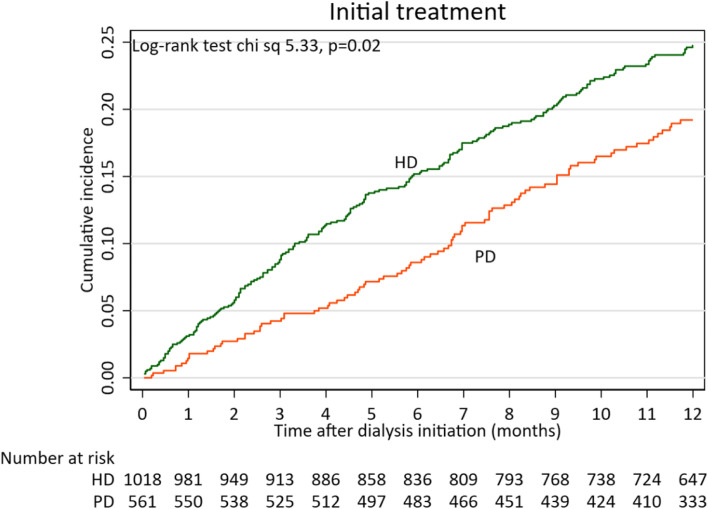
Kaplan Meier curves showing relationship of initial dialysis treatment (in-centre HD or PD) with first year all-cause mortality among 1580 patients starting on dialysis

**Fig. 2:**
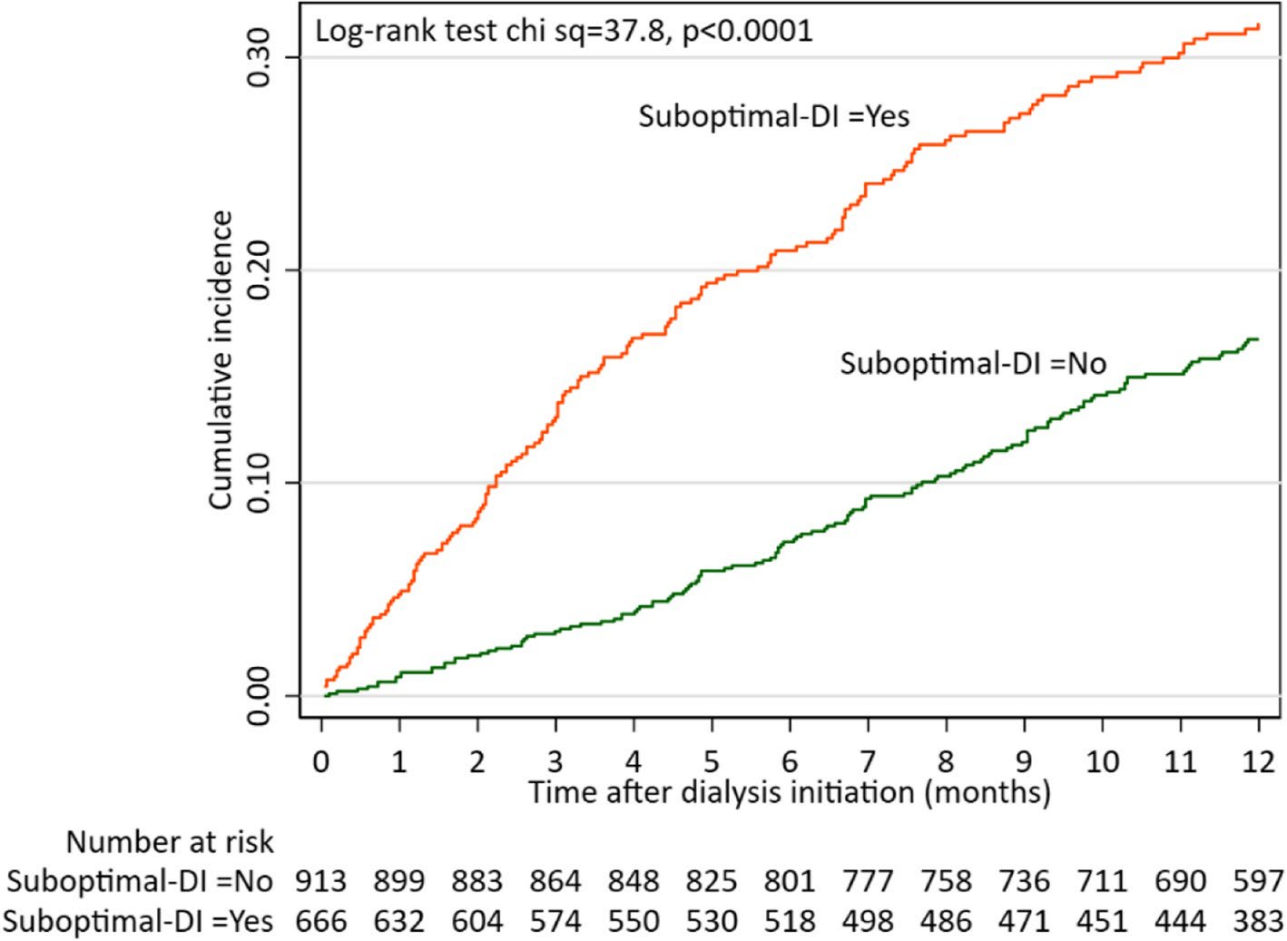
Kaplan Meier curves showing relationship of initial dialysis treatment with first year all-cause mortality among 1580 patients starting dialysis

The mortality rate (deaths per 100 patient-years) was 23.90% between 0 and 3 months and 19.33% between 0 and 12 months. There was no significant difference in mortality between the countries and regions involved in the study. The most important comorbid conditions were heart failure and peripheral atherosclerosis (12-months mortality rate, 36.7% and 35.3%, respectively). First year mortality rate was higher among patients starting on HD compared to the healthier patients who started on PD, 21.26% vs 15.95% **(**Table 1 and Fig. 1). Patients with suboptimal DI had almost twice higher mortality at 12 months as compared to those starting in a planned way, 27.8% vs 13.8% (Fig. 2).

Among all 1580 patients, 1061 (67.2%) patients could select dialysis modality based on their personal choice and were differentiated from patients with contraindications to one or the other dialysis modality. Of these 1061 patients, 654 (61.6%) chose PD, 368 (34.7%) in-center HD and 39 (3.7%) home HD. The number of home HD patients was too small and home HD patients were excluded from further analyses. Furthermore, among those who had chosen PD, 93 patients (14.2%) did not receive PD as their initial treatment, usually because they were started on HD, and 4 (0.1%) patients who had chosen in-centre HD did not receive this treatment.

Among the 1022 patients, who could select dialysis modality based on their personal choice and who could start on PD (*n* = 654) or in-centre HD (*n* = 368), PD patients had a lower incidence of late referral and unplanned DI and lower incidence of eGFR loss rate > 1 ml/min, but a higher eGFR at DI. There was no overall significant difference in mortality risk between PD choice as compared to HD choice patients**;** unadjusted hazard ratio, HR, 0.79 (95% CI 0.56–1.12), adjusted HR 0.81 (0.56–1.15). A subgroup analysis including only those patients who had a planned optimal DI (*n* = 691) also showed no significant difference between the two modalities; crude HR, 0.84 (95% CI 0.52–1.34), adjusted HR 0.82 (0.51–1.30).

**Fig. 3:**
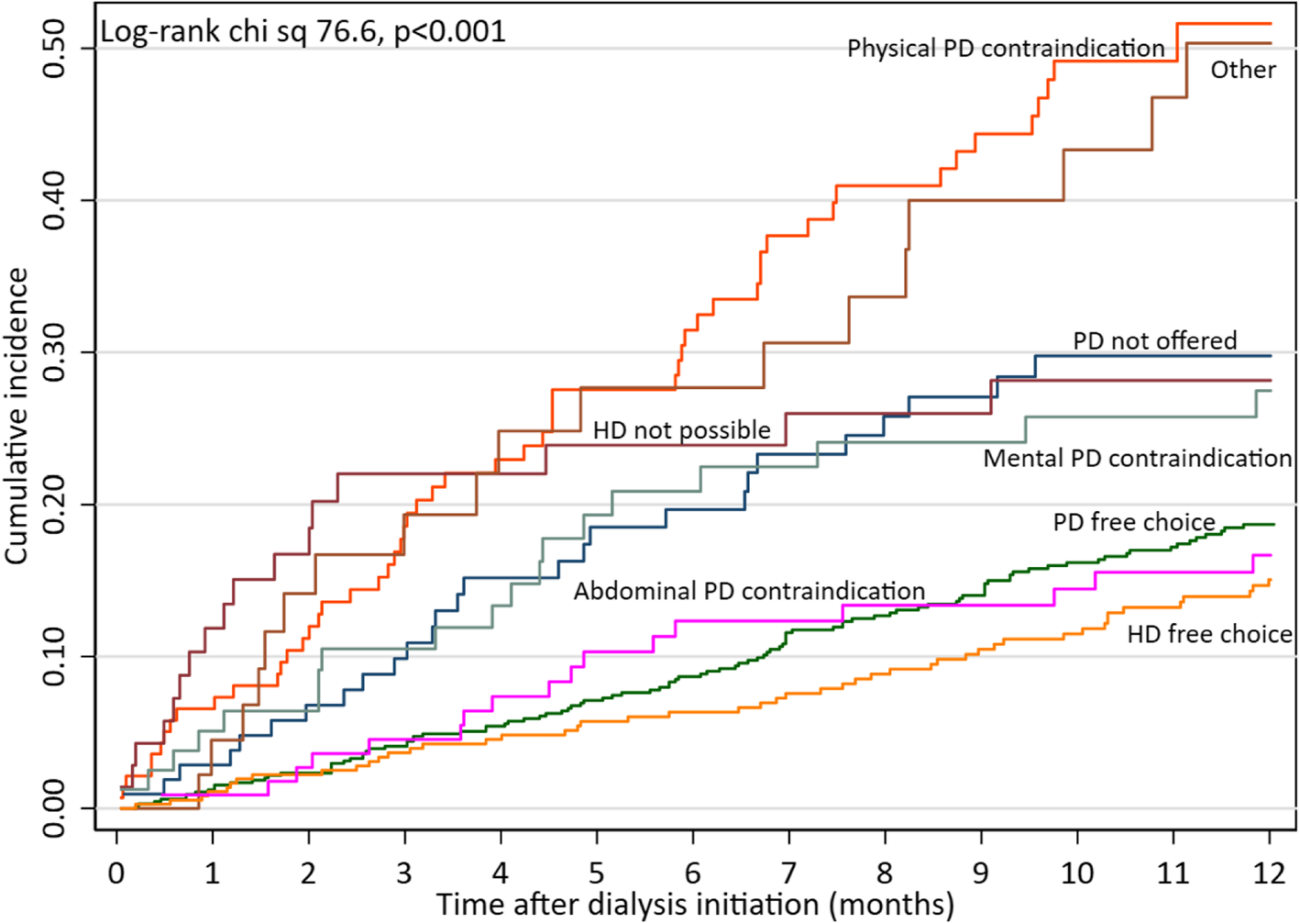
Kaplan Meier curves showing relationship between first year all-cause mortality and cause of initial modality choice among 1580 patients starting dialysis. At one year, mortality was highest (> 50%) among patients with Physical PD contraindication (*n* = 71) and patients with Other contraindications (*n* = 142); intermediary high (20–30%) for the categories PD not offered (*n* = 106), Mental PD contraindication (*n* = 80) and HD not possible (*n* = 46); and, lowest (15–20%) for patients with “free choice” of PD (*n* = 654) or HD (*n* = 368) and patients with Abdominal PD contraindication (*n* = 113)

First year cumulative mortality rates varied considerably depending on the eight registered categories of causes of modality choice (Fig. 3). At one year, mortality was highest (> 50%) among patients with Physical PD contraindication (*n* = 142) and patients with Other contraindications (*n* = 71); intermediary high (20–30%) for the categories PD not offered (*n* = 106), Mental PD contraindication (*n* = 80) and HD not possible (*n* = 46); and, lowest (15–20%) for patients with “free choice” of PD (*n* = 654) or HD (*n* = 368) and patients with Abdominal PD contraindication (*n* = 113).

**Fig. 4:**
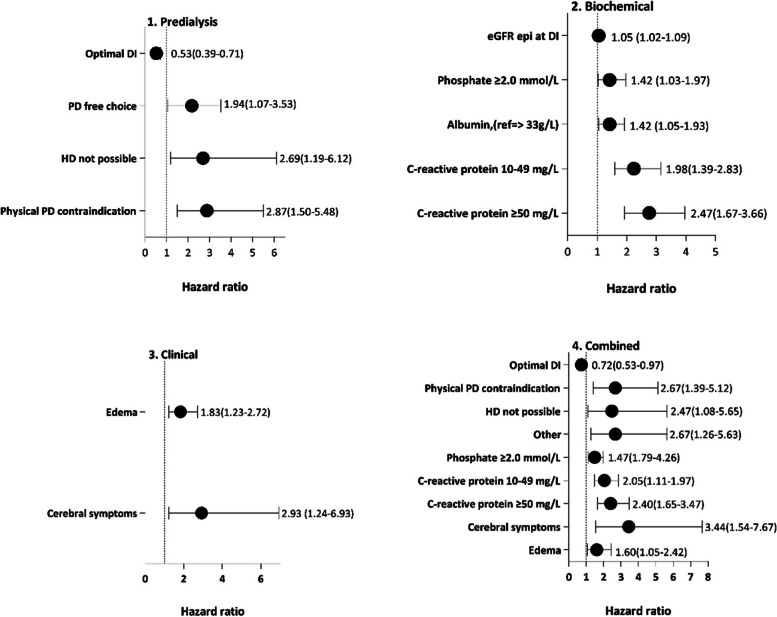
Forest plot showing significant associations of factors with first-year all-cause mortality risk among 1580 patients starting dialysis in three separate models and a combined model including factors with significant associations to mortality in the three separate models. Survival was analysed by a flexible parametric model with stpm2 command. Results are expressed as hazard ratios for all-cause mortality with 95% confidence interval. All models were adjusted for age, sex, renal diagnosis, and presence of comorbidity. For cause of choice, reference was "HD free choice"

We analysed factors associated with first year all-cause mortality in four models using a flexible parametric model with stpm2 command. All models were adjusted for age, sex, renal diagnosis, and presence of comorbidity. All other factors with significant associations, expressed as hazard ratios, with mortality are shown in Fig. 4. In the adjusted multivariate Predialysis model, suboptimal DI, physical contraindication to PD, contraindication to HD and PD free choice (the latter was compared with “HD free choice” which was associated with lowest mortality rate, see Fig. 3) were associated with increased mortality risk. Rapid eGFR loss 3 to 0 months before DI was borderline significant (*p* = 0.08). In the adjusted Biochemical model, high eGFR, high phosphate, low serum albumin and high CRP were associated with increased mortality. In the adjusted Clinical model, focusing on clinical problems linked to the primary cause of DI, edema, and cerebral symptoms were the only independent risk factors for death. Finally, in the Combined model, suboptimal DI, physical PD contraindication, HD contraindication (HD not possible), “other causes” of modality choice, hyperphosphatemia, inflammation (raised CRP), edema, and cerebral symptoms predicted higher risk of death independently of age, sex, renal diagnosis and registered comorbidity (Fig. 4).

## Discussion

In our study, factors associated with one-year mortality after DI among 1580 incident dialysis patients participating in the Peridialysis study [26–28] were investigated in three models that—in addition to age, sex, renal diagnoses and comorbidity—included data on predialysis course (“Predialysis” model), biochemical parameters (“Biochemical” model), and primary clinical cause of DI (“Clinical” model). A fourth “Combined model” that included all statistically significant factors in the three separate models, showed that suboptimal DI, physical PD contraindication, HD contraindication, Other contraindications, hyperphosphatemia, inflammation (elevated CRP), edema and cerebral symptoms were associated with increased risk of death within one year after DI, independent of age, sex, renal diagnosis and comorbidity (Fig. 4), whereas eGFR at DI or rate of loss of eGFR prior to DI was not related to mortality. These findings highlight the importance of suboptimal DI, a potentially modifiable factor, as a contributor to early mortality in patients starting on dialysis. In patients with a “free” choice of dialysis modality based on their personal preferences, PD and in-center HD led to broadly similar short-term outcomes (Fig. 3). Our results thus support current recommendations that modality choice should be made according to patient preference rather than for medical reasons [32, 33].

It should be noted that patients´ preferences are influenced by many factors. Social circumstances of patients may constrain their apparent "preferences” for one therapy over another because their mental and physical capacity are limited by the “burden of illness, either by the disease or its treatment (including medications or side effects from dialysis), and that access to and use of healthcare and the potential for self-care may be limited by inadequate patient capacity [34]. Variables reflecting psychosocial characteristics such as employment status and need for ambulatory assistance may influence patient´s choice in the decision to initiate a particular dialysis modality [35].

In a previous study of factors associated with suboptimal DI in the same population, we found that late referral and rapid eGFR loss were independent predictors of suboptimal DI and that patients with suboptimal DI were more uremic at DI as judged by eGFR, had more electrolyte disturbances over and above what would be expected from the level of eGFR, and had a higher CRP [28]. In the present study, suboptimal DI associated with markedly increased mortality rates (Fig. 2).

When analyzing independent predictors of increased mortality in the separate multivariate models, the model focusing on factors involved in the predialysis course (“1. Predialysis” model), showed optimal DI as compared to suboptimal DI associated with 47% reduction of mortality risk (HR 0.53, 95%CI 0.39–0.71)1. and that three causes of modality choice were associated with higher mortality risk (as compared to reference group HD free choice): PD free choice, HD not possible and PD physical contraindication.

In the multivariate analysis focusing on biochemical parameters (“Biochemical” model), a high eGFR at DI, hyperphosphatemia (> 2.0 mmol/L), low serum albumin (< 33 g/L), and elevated CRP (> 10 mg/L) associated independently with mortality. As discussed below, the finding that high eGFR associated with increased risk is conceivably due to patients with complications being started earlier at higher eGFR and that in patients with lower muscle mass, serum creatinine is lower and inflates eGFR. Finally, in the model focusing on clinical causes of DI (“Clinical” model), only edema and cerebral symptoms (primarily coma) associated with increased risk.

Some of these findings have previously been reported. Causal explanations of these associations are purely speculative. Many patients had what were perceived to be contraindications for PD including previous abdominal surgery, other physical reasons such as polycystic renal disease, and “mental” contraindication meaning that they were not considered to be capable of self-dialysis, and some patients had contraindications for HD such as problems to get vascular access. It should be noted however that while there are few absolute contraindications to PD, physicians may regard (rightly or wrongly) factors such as advanced age, massive comorbidity burden, obesity, polycystic kidney disease, heart failure, and previous history of abdominal surgery and renal allograft failure, as relative contraindications to initiation of PD [36]. It is possible that the coded contraindications could introduce selection bias in determining use of modality and representation in this study's sample. Physical PD contraindication and HD contraindication can be regarded as a surrogate marker of comorbidity. One can imagine that an acute infection, e.g., pneumonia, could result in accelerated eGFR loss and suboptimal DI, the pneumonia being the primary cause of death. An alternative scenario emphasizes the pre-dialytic course, where rapid eGFR loss leads to delayed dialysis planning, with subsequent requirement for suboptimal DI in a clinical situation of severe uraemia, pulmonary stasis, and acute infection. Subsequent mortality would then be a consequence of poor clinical condition at DI and subsequent catheter-related complications.

Rapid loss of renal function prior to DI has previously been associated with mortality [15, 37] and could be expected to continue after DI. While rapid eGFR loss predicted suboptimal DI [28], it had an only a marginal non-significant effect on subsequent mortality in the present study.

The presence of significant residual kidney function is reported to be associated with reduced mortality after DI, independently of total (renal and dialysis) Kt/V or creatinine clearance [38, 39]. In contrast, we found that high rather than low eGFR was associated with mortality, and several large epidemiological studies have also shown a paradoxical inverse relationship between eGFR at DI and subsequent survival [40–42]. It has been suggested that this is due to patients with high comorbidity starting dialysis at a higher eGFR [42] or that eGFR is artificially raised in cachectic patients with a low muscle mass [43]. Altogether these observations are in accordance with the IDEAL study [44], a randomized controlled trial which found no difference between early and late DI while in a study in Swedish dialysis patients very early initiation of dialysis was reported to be associated with a modest reduction in mortality and cardiovascular events in Swedish dialysis patients [45]. Our study thus suggests that DI should primarily be based on clinical rather than biochemical indications, with particular attention paid to patients with a rapid loss of renal function, which may lead to suboptimal DI [26].

Many studies have been published concerning the relative survival of PD and in-center HD patients including recent comprehensive reviews [33, 46, 47]. Most studies show a reduced mortality of PD relative to HD during the early period after DI [48–56], but others an increased mortality [12, 47, 57, 58] or no difference [59–61]. PD seems to be advantageous for younger patients and non-diabetics. There are plausible reasons why this difference may be causal. PD is associated with less hemodynamic stress, and a slower loss of residual renal function, a factor which is generally recognised to improve health and prognosis [62, 63]. Hemodialysis is associated with a marked initial acceleration of mortality, particularly cardiovascular [64–66], that may be due to the unphysiological fluctuations in solutes and fluid and the cardiac strain of hemodialysis that increase the risk for sudden cardiac death, a common cause of death in dialysis patients [5, 67, 68]. On the other hand, studies comparing the mortality of patients on peritoneal dialysis (PD) versus hemodialysis (HD) are complicated by the fact that patients are more likely to be treated with HD if they have multiple comorbidity and suboptimal DI, usually defined as unplanned DI in an in-patient setting using a temporary central venous catheter [69], and therefore statistical adjustments need to be performed. However, it has been suggested that the apparent early PD survival advantage is due to previous statistical analyses not correcting sufficiently for differences in patient clinical status, and that there are no major differences in mortality [59, 60, 70].

A major advantage of this study, compared to other studies, is that patients were assessed before or at DI for suitability for HD and PD. This has not previously been studied as a confounding factor. Excluding unsuited patients will result in a more accurate assessment of the consequences of choosing in-center HD or PD. As previously described [14], PD patents had a slower rate of eGFR prior to DI and had a higher eGFR at DI, but this did not affect the finding regarding mortality. Our results support the hypothesis that there are no major differences in short term prognosis for incident patients who were able to make a free choice based on their personal preference (Fig. 3).

This study has several limitations that should be considered when interpreting the results. Being an observational study, causal conclusions cannot be made. The number of patients is insufficient to allow reliable sub-group analysis and the study was not designed to demonstrate differences in mortality between HD and PD patients. Furthermore, we did not apply correction for multiple testing and thus the results should be regarded as purely descriptive. While multidisciplinary predialysis care and use of shared decision making with involvement of patients and their family and the renal team was routine in the centres involved in this study [26–28], the quality and quantity of this care and the education or guidance provided to patients were not assessed. Potential implications of coded contraindications in terms of introducing selection bias in determining use of modality and representation in this study's sample were not considered. As suboptimal DI and late referral are overlapping in many cases, we did not analyze separately the possible impact of late referral on subsequent mortality. Finally, the physicians who reported these data may have differed in their assessments of patient modality suitability.

In conclusion, first-year mortality in incident dialysis patients was—in addition to high age and comorbidity—associated with suboptimal DI, contraindications to one or the other modality, hyperphosphatemia, inflammation, edema, and cerebral symptoms while eGFR at DI or rate of loss of eGFR prior to DI did not appear as independent predictors. These findings highlight that suboptimal DI, a potentially modifiable factor, is a significant contributor to early mortality in patients starting on dialysis, indicating that predialysis care, early education and planning are warranted as it is possible that an intensive pre-dialytic program, with early access planning, and particular attention paid to rapid eGFR loss, infectious complications and overhydration prophylaxis could reduce early mortality after DI. Our results that PD and in-center HD led to broadly similar short-term outcomes, support current recommendations that modality choice should be based on patient preference rather than medical reasons only [32, 33].

## Data Availability

Data and materials from this study can be obtained from the corresponding author. The data is available on Open Science Framework osf.io/3wh4g.
